# Assessing social recovery of vulnerable youth in global mental health settings: a pilot study of clinical research tools in Malaysia

**DOI:** 10.1186/s12888-019-2164-x

**Published:** 2019-06-20

**Authors:** Clio Berry, Ellisha Othman, Jun Chuen Tan, Brioney Gee, Rory Edward Byrne, Joanne Hodgekins, Daniel Michelson, Alvin Lai Oon Ng, Nigel V. Marsh, Sian Coker, David Fowler

**Affiliations:** 1 School of Psychology, Pevensey I, University of Sussex, Falmer, Brighton, East Sussex BN1 9QH UK; 2 Research & Development, Sussex Partnership NHS Foundation Trust, Sussex Education Centre, Millview Hospital, Nevill Avenue, Hove, BN3 7HY UK; 3 SOLS HEALTH, SOLS 24/7, 1Petaling Commerz and Residential Condos, #G-8, Jalan, 1C/149, Off Jalan Sungai Besi, Sungai Besi, 57100 Kuala Lumpur, Malaysia; 4 Clinical Psychology, Norwich Medical School, University of East Anglia, Norwich Research Park, Norwich, Norfolk NR4 7TJ UK; 5 Research & Development, Norfolk & Suffolk NHS Foundation Trust, 80 St Stephens Road, Norwich, NR1 3RE UK; 6 Psychosis Research Unit, Greater Manchester Mental Health NHS Foundation Trust, Psychosis Research UnitHarrop House, Prestwich Hospital, Bury New Road, Manchester, M25 3BL UK; 7 Department of Psychology, Faculty of Science and Technology, Sunway University, No. 5, Jalan Universiti, Bandar Sunway, Petaling Jaya, Selangor Malaysia; 8 Department of Psychology, James Cook University, 149 Sims Drive, Singapore, 387380 Singapore

## Abstract

**Background:**

A social recovery approach to youth mental health focuses on increasing the time spent in valuable and meaningful structured activities, with a view to preventing enduring mental health problems and social disability. In Malaysia, access to mental health care is particularly limited and little research has focused on identifying young people at risk of serious socially disabling mental health problems such as psychosis. We provide preliminary evidence for the feasibility and acceptability of core social recovery assessment tools in a Malaysian context, comparing the experiential process of engaging young Malaysian participants in social recovery assessments with prior accounts from a UK sample.

**Methods:**

Nine vulnerable young people from low-income backgrounds were recruited from a non-government social enterprise and partner organisations in Peninsular Malaysia. Participants completed a battery of social recovery assessment tools (including time use, unusual experiences, self-schematic beliefs and values). Time for completion and completion rates were used as indices of feasibility. Acceptability was examined using qualitative interviews in which participants were asked to reflect on the experience of completing the assessment tools. Following a deductive approach, the themes were examined for fit with previous UK qualitative accounts of social recovery assessments.

**Results:**

Feasibility was indicated by relatively efficient completion time and high completion rates. Qualitative interviews highlighted the perceived benefits of social recovery assessments, such as providing psychoeducation, aiding in self-reflection and stimulating goal setting, in line with findings from UK youth samples.

**Conclusions:**

We provide preliminary evidence for the feasibility and acceptability of social recovery assessment tools in a low-resource context, comparing the experiential process of engaging young Malaysian participants in social recovery assessments with prior accounts from a UK sample. We also suggest that respondents may derive some personal and psychoeducational benefits from participating in assessments (e.g. of their time use and mental health) within a social recovery framework.

## Background

Although the majority of adolescent populations reside in low and middle-income countries (LMICs), little research has focused on the identification, prevention and treatment of serious and socially disabling mental health problems in these countries. A recent priority-setting exercise for global child and adolescent mental health research [1] highlighted the dearth of evidence on early intervention for psychosis in LMICs, with only one identified trial from China and few cross-cultural validations of screening tools. Psychosis tends to first occur during adolescence and is a leading worldwide cause of disability; with social disability often observed before, during and after the first psychotic episode [2–5]. The first episode of psychosis – and the preceding ‘prodromal’ period – represent key opportunities for early intervention [6–10]. The provision of evidence-based early intervention services globally is very variable, however, and standard care for psychosis rarely meets the minimum standards suggested by the World Health Organisation’s (WHO) Mental Health Gap Action Programme [11]. Access to care in LMICs typically lags far behind the first onset of symptoms [12, 13], which increases risk for poor long-term prognosis [14]. Thus, identifying and intervening early for young people who are at risk for serious, socially disabling mental health problems – and especially transition to psychosis – remain critical yet largely neglected challenges in LMICs.

In Malaysia, access to mental health care is particularly limited. Malaysia is a Southeast Asian country of 32 million people. The majority ethnic group is Bumiputra (68%), comprising a majority of Malays and a minority of other indigenous people [15]. The other major ethnic groups are Chinese (23%) and Indian (7%) [15]. Malaysia is a Muslim-majority country but many people identify as Christian, Buddhist, Hindu, Taoist, Sikh and other minority religions [16]. Epidemiological estimates suggest that mental health problems in Malaysia have more than doubled over the last 20 years and now affect at least 30 to 40% of the adult population [17, 18]. Young people aged 16 to 24 years are particularly at risk of developing mental health problems [18]; the estimated prevalence of youth mental health problems in Malaysia exceeds the average worldwide prevalence [19] and may be increasing [20]. Amongst young Malaysians, people from low-income and/or indigenous backgrounds show increased vulnerability to mental health problems [18, 20]. The mean average Duration of Untreated Psychosis (DUP) in Malaysia is over 3 years, which has significant negative implications for prognosis [13].

Where available, Malaysian mental health services are largely based on Western models of psychiatry and clinical psychology [21]. There is evidence that Western models may have broad application, with positive impacts evidenced in South East Asia and Malaysia specifically [21–23]. Nevertheless, the universality of Western approaches remains largely untested in the local context [21, 24]. The relative importance of communality and collectivism in the Southeast Asian cultures [24] may also complicate the ‘fit’ of Western approaches which foreground individual support and self-enhancement [25, 26]. Moreover, the cultural validity of Western approaches in serious mental health problems is further complicated by differences in understandings of unusual experiences or psychosis [24, 27] and significant heterogeneity of health belief systems amongst different ethnic groups in Malaysia [27]. Thus, whilst Western approaches may benefit the development of psychological interventions in Malaysia, exploring the cultural validity of such approaches prior to and during implementation is essential. For example, this may reveal potential clashes of culturally determined values with imported therapeutic models and practices and could suggest scope for adaptation or optimisation through integrating Western approaches and Eastern philosophies [28, 29] — or else highlight a need for ‘bottom-up’ approaches grounded in the local setting [21].

A social recovery approach may have particular utility in Malaysia and other global mental health settings, where the social dimensions of serious mental health problems may be particularly poorly served. People accessing community-based rehabilitation services report extremely limited social support [30] and have highlighted their needs for interventions focused on increasing self-agency, social connections, social support and around increasing contact with and acceptance from the broader community [31]. Moreover, vocational support is rarely available in this part of the world [32]. There is preliminary evidence from Hong Kong that ‘case managers’ can provide social support and help facilitate socio-occupational functioning in schizophrenia [33], yet most practitioners in Malaysia lack adequate training and experience in working with individuals with complex mental health and social needs [30].

Social Recovery Therapy (SRT; 5) may be a particularly promising intervention for the Malaysian—and broader LMIC—setting due to its focus on social recovery through personally meaningful and valued structured activity including employment, community, leisure and social activities. SRT is guided by personalised goals and values and gives specific attention to the individual’s wider context, and particularly their social networks [8, 34]. The intervention is informed by psychosocial constructions of mental health and recovery rather than a Western bio-medical model of mental ‘illness.’ As such, SRT is not primarily focused on diagnosis and symptom reduction; rather symptoms are attended to only insofar as they form barriers to social recovery (in addition to other personal and systemic barriers of relevance). In the UK, SRT has been found to be an effective treatment for young people experiencing social disability following psychosis [34, 35] and is currently being tested for young people with complex emerging mental health problems including at-risk mental states for psychosis [7]. Moreover, SRT provides practitioners with an explicit theoretical framework, manualised intervention procedures, and a set of therapeutic and assessment tools to facilitate patients’ social recovery. A clear framework and structured materials have been highlighted as important practice facilitators in previous research involving non-specialist mental health workers in high-income countries [36] and LMICs [37]. In addition, SRT recognises the contextual and cultural dependence of recovery and supports patients to formulate personally meaningful goals which are in line with their values [6].

The ‘fit’ of Western assessment tools needs to be explored in order to provide a foundation for applying a social recovery approach across diverse contexts. Qualitative accounts of using such tools are available from participants in a UK randomised controlled trial of SRT for 16–25 year olds with persistent social disability and complex emerging mental health problems [38, 39]. Participants identified positive aspects of disclosure and talking about difficult experiences during screening and outcome assessments [38, 39]. Participants also spoke of the benefits of exercises conducted within SRT in helping them to understand and manage barriers to structured activity [38]. Whilst these qualitative accounts support the acceptability of the social recovery approach in the UK, its suitability in other contexts is unknown. There is evidence regarding the semantic equivalence, validity, and reliability of some social recovery assessments with relevant populations, for example measures of at-risk mental states for psychosis in Chinese populations [40]; however, the majority of relevant tools are untested outside the UK.

Our aim was to extend our prior qualitative work in the UK [38, 39] by piloting key social recovery assessment tools with young people in Malaysia, focusing on feasibility (i.e. whether the social recovery tools were easily, conveniently and successfully administered to participants; 31) and acceptability (i.e. whether the tools were favourably received by participants; 31). Feasibility was operationalised as time taken to complete assessment measures and rates of participant completion. Acceptability was examined with respect to the qualitative experiences of participants, using a deductive coding framework derived from our prior work in the UK [38, 39].

## Methods

### Design

We performed a cross-sectional pilot study to assess young people’s experiences of undertaking a multi-faceted structured assessment of their mental health and social disability.. The focus was on the experiential process of completing existing standardised social recovery measures that would be completed as part of a clinical research assessment, i.e. the assessment of time use, unusual psychological experiences (e.g. hearing voices), emotional problems, and positive and negative self-beliefs [7]. We focused additionally on the completion of assessments typically used within the therapeutic assessment and formulation process conducted within SRT therapy; i.e. a values assessment and social identity mapping exercise. We also incorporated a more generic youth mental health screening and outcome measure as potentially more viable to capture emotional problems as part of a social recovery approach in Malaysia compared to more technical and resource-intensive diagnostic assessments used in the UK [7].

### Participants

Following ethical approval from the University of Sussex (Reference: CB/321/8) and relevant local approvals, a sample of participants were recruited from a non-government educational and mental health social enterprise and partner organisations in Peninsular Malaysia. Inclusion criteria required participants to be between 16 and 30 years old, able to provide informed consent, and be vulnerable young people under the institutional care of a Non-Government Organisation (NGO) in a full residential setting. The NGO and partner organisations serve low-income populations (defined as earning 40% less than the national average) in crime-affected localities in greater Kuala Lumpur. The low socio-economic status of the vulnerable target population also manifests as a lack of access to basic services such as housing and formal education. The organisations included orphanages which serve young people who are unable to remain in the family home due to extreme poverty, neglect and/or trauma. Participants did not need to report experiencing previous or current mental health problems to participate.

Potential participants were first approached by NGO staff members. Consent from the parent or caregiver with parental responsibility was sought before approaching potential participants aged under 18 years old. Interested young people were provided with information about the study. After obtaining verbal agreement for contact from the study team, each participant was invited to meet for an interview with a researcher and an interpreter. Participants were sampled using convenience sampling approach that maximised ethno-cultural diversity across Indigenous (Orang Asli), Malay, Chinese and Indian participants and the three primary languages of Malay, Mandarin and Tamil. The final sample (*N* = 9) comprised 5 males and 4 females, aged 16 to 23 years (M = 19.78 years; SD = 2.86). No participants reported a diagnosis of physical or mental health problems. Four participants were referred by the social enterprise, 2 from a partner educational organisation and 3 from a looked after children’s home or orphanage. All invited participants had at least 8 years of formal education. No approached participants declined. One additional orphanage was approached but did not refer any potential participants, with reasons unknown. An additional young person who was referred was not invited to consent due to having a serious learning disability which precluded capacity to provide informed consent.

### Experiential process measures: social recovery clinical research assessments

#### Time use survey (TUS)

The TUS is a validated semi-structured interview measure of time use in clinical and non-clinical populations [9], derived from an Office for National Statistics (UK) survey [41]. Respondents recall time spent in structured activities over the past month (paid and voluntary employment, education, housework, childcare, sports, and leisure) which is then averaged into weekly hours.

#### Prodromal questionnaire (PQ-16)

The PQ-16 [42] is a 16-item true/false self-report questionnaire. A score of 6 or more indicates elevated risk of psychosis. There is evidence of good validity and reliability in a Chinese population [40].

#### Comprehensive assessment of at risk mental states (CAARMS)

The CAARMS [43] is a semi-structured interview capturing intensity, frequency and duration of subthreshold psychotic symptoms. Scores across unusual thought content, non-bizarre ideas, perceptual abnormalities, and disorganised speech subscales, plus Global Assessment of Functioning (GAF) scores were used to determine At Risk Mental States (ARMS) status. There is evidence of good reliability and validity in a Japanese population [44].

#### Brief Core Schema scales (BCSS)

The BCSS [45] is a 24-item self-report measure in which participants rate agreement with 6 positive and 6 negative beliefs about themselves and other people from 0 (No) to 4 (Believe totally). The BCSS has been used successfully in Japan and Indonesia [46, 47].

#### Strengths and difficulties questionnaire (SDQ) adolescent version

The SDQ [48] is a 25-item brief behavioural screening questionnaire designed to identify emotional and behavioural problems. Participants rate item agreement as Not true, Somewhat true, or Certainly true. Many translated versions of the SDQ exist - including a Malay parental informant version; however there is limited information about linguistic or semantic equivalence [49]. Nevertheless, completion by Malay parents of either the Malay or English version of the questionnaire has been found to have negligible impact on the scores [49].

### Experiential process measures: SRT therapeutic assessment tools

#### Social identity map (SIM)

The SIM tool [50] produces a visual representation of participants’ social groups. After identifying all their social groups and rating each group’s importance from 1 (not at all important) to 5 (very important)), participants rate number of days actually spent with the three most important groups in the past month (0 to 30), number of days that they would have liked to have spent with these groups (0 to 30), and inter-group compatibility (easy, moderately easy, and hard).

#### Values assessment

The Values Assessment is an adaptation of the Valued Living Questionnaire [51], in which participants state valued directions for each of ten life areas, for example, employment. Participants then rate from 1 to 10 (least to most) the absolute importance of each valued direction and how consistently they are living in accordance with the valued direction. Finally, participants rank the valued directions from 1 to 10 according to their relative importance.

### Feasibility and acceptability

Feasibility was first assessed by recording the time taken to complete the assessments and rates of completion. In order to evaluate acceptability, a semi-structured interview schedule was derived from the PRODIGY trial schedule [38, 39]. We retained questions regarding experiences of completing the research assessments and removed questions relating to specific PRODIGY trial procedures. We added specific questions to explore the process of completing the assessments, for example; “What was it like for you when we asked you about social groups that you belong to?”

### Procedure

After providing written informed consent, participants engaged in a combined assessment and interview session conducted by the first author in the presence of an interpreter. Sessions were conducted in a private location convenient to the participant; in clinic or meeting rooms on NGO premises, in the participant’s home or place of work. The duration of the assessments is reported below. Qualitative interviews lasted between 16:53 and 41:31 min (*Mean* = 26:20, *Standard Deviation* = 8:23). Interpreters (*N* = 6; 5 female and 1 male) were staff members (therapists and/or programme directors) from the mental health arm of the collaborating NGO to allow for signposting and provision of support services to participants if necessary. Interpreters had received a one-day training session on the study aims, social recovery approach, and assessment procedures. Assessments were not translated in advance but were administered by the first author in the English language. Interpreters provided interpretation as needed for participant comprehension. The interviewer checked understanding of interpreted questions and responses with all parties through further questioning and additional interpretation was conducted as needed. Interpreters variably used first, second, or third person pronouns within and across interviews. For ARMS screening purposes, all participants were asked to complete the PQ-16 and any participant scoring 6 or more was asked to then complete the CAARMS assessment. All sessions were audio-recorded using a digital recorder with participant permission and the English content was transcribed verbatim.

### Qualitative analysis

A deductive thematic analysis [52, 53] approach was used to cross-validate themes identified in the previous UK PRODIGY studies [38, 39]. The thematic analysis was conducted using six of Braun & Clarke’s seven steps [53]; transcription, familiarisation, coding, searching for themes, reviewing themes, and defining themes. The seventh step, naming themes, was not performed. Coding focused on coding units of text which appeared to reflect the presence of themes from the previous accounts. Searching for and reviewing themes focused on reviewing the ‘fit’ of present data with these previous themes, analysing the thematic content of the coded excerpts, and identifying manifestations of the respective ‘central organising concepts’ [53]. These steps also involved re-reading and re-familiarisation with the previous themes [38, 39] to ensure continual reflection on the ‘fit’ of present data. At least two authors independently coded 80% of transcripts to ensure reliability in coding and identified themes.

## Results

### Feasibility

Descriptive statistics are provided to contextualise the sample (Table 1). Assessments lasted between 53 min and 58 s and 2 h, 11 min and 10 s (Mean = 1:20:15, Standard Deviation = 32:05).

**Table 1:** Quantitative assessment and therapeutic tool descriptive statistics and rates of participant non-completion

	N(%)	M(SD)	Range	Non-completion N(%)
Time Use Survey (TUS)	9 (100)			0
Structured activity		52.74 (19.34)	21.26–80.46	
Unstructured direct socialising		5.97 (8.51)	0–26.77	
Unstructured indirect socialising		20.22 (23.53)	0–70	
Prodromal Questionnaire (PQ-16)		5.11 (3.62)	1–11	0
Proportion scoring 6 plus	4 (44.44)			
Comprehensive Assessment of At Risk Mental States (CAARMS)	3 (33.33)			1 (25)
Scoring At Risk	1 (33.33)			
Scoring Not at Risk	2 (66.67)			
Brief Core Schema Scale (BCSS)				1 (11.11)
Positive self		12.44 (6.06)	0–19	
Negative self		5.89 (4.59)	0–16	
Positive other		13.89 (8.21)	0–22	
Negative other		7.22 (8.09)	0–21	
Strengths and Difficulties Questionnaire (SDQ)		14.90 (8.05)	0–24	1 (11.11)
Social Identification Map (SIM)				2 (22.22)
Number of social groups identified		4.57 (0.79)	3–5	
Importance of groups identified		4.66 (0.54)	3–5	
Actual days spent with groups in past month		16 (12)	0–30	
Ideal days spent with groups in past month		17 (12)	0–30	
Values Assessment				2 (22.22)
Number valued directions identified		9.71 (0.49)	9–10	
Importance of valued directions		10 (1)	7–10	
Current success in valued directions		7 (3)	0–10	

Rates of participant completion are shown in Table 1, with the lowest rate of completion at 75% for the CAARMS. Reasons for non-completion are as follows. One participant was not invited to complete the CAARMS where indicated due to researcher concerns regarding participant fatigue and comprehension. One participant did not complete the BCSS due to another commitment. Two participants completed neither the SIM nor the Values Assessment; one participant requested to finish the assessment due to fatigue and other commitments, the other participant wished to end the assessment session due to fatigue and discomfort related to especially warm weather. The latter participant also did not complete the SDQ.

### Acceptability: part one

We first assessed cross-validation of current data against the five themes identified in the first qualitative study from the PRODIGY trial [39]; ‘Practicalities’, ‘Acceptance’, ‘Disclosure’, ‘Altruism’ and ‘Engagement’.

#### Practicalities

This theme related to practical aspects of completing assessments. One participant commented on the duration of the assessments; “*I think the time… maybe they* [other participants] *don’t have too long… for me is okay but I don’t know* [about] *other people*” (Participant 5). Another participant suggested that “*You could put it in tablet* [computerised] *form… maybe they could answer themselves… quicker, yeah*” (Participant 2), but also recommended increasing the scope to assess time use over three to 6 months instead of the standard previous one-month reference period:

“*… maybe you can make the timeline longer, not just past month… three to six months… maybe sometimes we are just busy with one thing in past month and then we didn’t do much things…. longer period… that would be more accurate maybe*” (Participant 2).

Being asked to accurately recall activity during the past month was challenging for some people; “*hard to remember*” (Participant 4).

#### Acceptance

This theme was identified with respect to overall acceptance of sensitive assessment questions albeit with some less positive experiences. One participant stated; “… *because you just asked me ‘How long am I in the restaurant?’, like police*” (Participant 5), thus perhaps experiencing the time use assessment to be somewhat repetitive or interrogatory. Three participants expressed responses to SDQ assessment questions which could reflect mild discomfort. For two participants this related to revisiting difficult memories; “*When answer I-I really a lot think of what kind of things that I worry, that feels… flashback yeah*” (Participant 9), “*I will remember about the past past story in myself before I-I came here, yeah, so I feel like not really okay*” (Participant 3). The third participant reported nervousness when being asked about their mood, which they suggested was related to perceiving the interviewer to be in a position of authority; “… s*o when you’re asking the question it makes him feel like you are in an authority position… like a higher position.*” (Interpreted, Participant 1). Being asked the same questions by someone known to the participant was seen as a way to potentially mitigate discomfort; “*Maybe nervous, but maybe better, but not-not as much, not as much as if it’s someone else.*” (Interpreted, Participant 1).

All participants nevertheless reported finding the assessments to be at least acceptable, if not beneficial, overall; “*I feel comfortable… I feel like that is helpful for me for me to answer and to know about my activities, everything yeah*” (Participant 3). All participants indicated willingness to complete the same assessments again in the future. Four participants suggested that even difficult questions could be helpful, for example; “*It’s I think helpful… I always thought about this and sometimes I feel like I want to change my mind… because before, before and now, I can compare*” (Participant 3).

Participants also emphasised the novelty and strangeness of being asked about unusual psychological experiences as part of the PQ-16 and CAARMS; “*He kind of feels like a little bit strange… because for him it is very unfamiliar to him, asking the questions… because I never encounter these kind of questions before*” (Interpreted, Participant 2). Two participants likened questions about unusual experiences to horror films:

“*Actually yesterday I just watched* [a horror film] *then the questions that you asked, this one make me remember the scene where was quite scary… The sounds, these two the most scary, ‘I have seen the face change right* [right before my eyes’ PQ-16 [42]]*’, so is like the movie, is creepy*” (Participant 9).

“*The clapping, the hissing … like he, so he always watch … what kind of movie? … Scary movie. Yah. So like these kind of things always in the movie*” (Interpreted, Participant 6).

However, all participants described these questions as fully acceptable.

#### Disclosure

The theme of disclosure was represented across participant accounts. Three participants expressed some reticence or concern around disclosing emotional problems or mental distress, for example; “*It feels like a bit nervous… like the thing that I want to tell… like say really embarrassed*” (Participant 8). For another participant, disclosure was dependent on perceptions of privacy and trustworthiness - for example of the researcher; “*I can tell you, I-I can believe you can keep my secret… very hard to ask these questions… but I can trust you*” (Participant 4). Reticence in disclosing emotional distress appeared more evident for participants from looked after children’s homes (orphanages) although benefits were also identified: “*I feel good … If you want to come back, then I can see you. If you, next day, if you come back I can tell you everything*” (Participant 4).

#### Altruism

Altruism as a motivation for research participation was not explicitly identified in the present sample. As the current work was not aligned to an intervention effectiveness trial, the projected future benefits of the study were perhaps less explicit. One participant, however, did emphasise the potential scientific value of this pilot research project, especially in relation to asking questions about unusual experiences which were perceived to be especially novel: “*… like asking questions that we never encounter before, so maybe you can find new discoveries… yeah and that’s good*” (Participant 2).

#### Engagement

Engagement was identified in the present study in relation to benefits derived from engagement in the therapeutic assessment tools. Engaging in the social map and values assessment was not without challenges. For example, one participant identified some potential discomfort in completing the social mapping exercise - in particular, quantifying the time spent with the identified social group relating to his faith:

“*With the example of the question on religion… difficult, is difficult to quantify whether is one time or three times*… *yeah because for me to answer… because asking if I want to challenge or be different than what is required…* [is] *disrespectful*” (Interpreted, Participant 1).

Quantifying time spent with his family group was also difficult; “*He remembers then, he remembered he had not been spending time with the family*” (Interpreted, Participant 1).

Nevertheless, all participants identified benefits associated with engaging in these therapeutic assessment tools. Participants suggested that being asked about their social groups and values was a very novel experience – and one that brought about increased self-awareness with regards to understanding their own values:

“*… before this, the people didn’t ask me about this and now, …I can answer my questions about this… the people also can know about my dreams*” (Participant 3);

“… *before this he never think about this and then after you wrote it down and then asked him about the importance, now he already think like what is… his achievement on… each of the… now his in this level and now in this level*” (Interpreted, Participant 7).

“[It helped him to think about] *how to educate or nurture his children okay how to have a happy family okay… how to help others… how to spend time with his family and friends… and plan. Good thing*” (Interpreted, Participant 1).

Many participants also reflected on the broader educational value of engaging in the overall process of completing assessment tools:

*"Experience… appreciating and helping... psychology …. learning… appreciating* (Participant 6); “[He] *really appreciate what he learns today because he like… as he is not very good person and not that educated… so he thinks is very valuable experience to know and learn this and all these things today*” (Interpreted, Participant 6).

There appeared to be something particularly enlightening about engaging in discussions about their unusual psychological experiences. Participants appeared to find these discussions normalising; “*I think for me now, now that you say to me those things, I feel like I could share with other people*” (Participant 8). Participants also seemed to suggest that asking people about unusual psychological experiences could facilitate sharing and open discussions with others, both within and outside of assessment type scenarios, and help facilitate people’s self-awareness regarding their own experiences:

“*At first they will share to you first and then they will ask whether he* [the participant] *experienced it or not. So when people share their experiences… so then suddenly then his feelings,his feelings on these things came… so people did ask that after they shared their experience*” (Interpreted, Participant 6).

“*Because this question I didn’t erm hear before and now I-I can, I can learn a bit from this questions… I can feel uh these questions like helpful for me… because I can, I can, I can remember that every day, we do, we can, we can feel like this. But some of these questions we didn’t, we didn’t feel but that is helpful for-for me to answer is like-like this ones I have seen here and… the sounds like banging, creaking… I can answer it even though I didn’t know*.” (Participant 3).

Additionally, questions about unusual experiences could facilitate an increased ability help support and signpost others: “*… since he already know this kind of questions, he know some of the symptoms… so if he knew he or someone else has it, so he will… they will… he will go straight to find someone… seeking help… go to psychologist or another person*” (Interpreted, Participant 7).

### Acceptability: part two

The three over-arching themes from the second UK PRODIGY SRT study [38], represented across participants in both SRT and treatment as usual arms of the trial, were then used for cross-validation: ‘“*It’s just the speaking to someone*”; the value of talking’; ‘“*Just do it*”; the importance of activity’; ‘Motivation for change’.

#### ‘It’s just the speaking to someone’; the value of talking

Attitudes regarding the value of talking were mixed. Some participants espoused benefits of talking to the researcher; “*I feel good*” (Participant 4). For other participants, the research assessment provided a valued space for self-reflection:

“*… because with such question I really think about whether like ‘Vulnerable? Am I vulnerable?’ So I really think about this question… I never thought about this… yeah I didn’t really spend time with myself yeah… probably because I always hang out with my friends, family, then travelling with friends, so I rarely have time to spend alone*” (Participant 9).

The ‘It’s not boiled up in me no more’ subtheme [38], was identified in accounts in which participants reported a stress-relieving effect of talking; “*Yeah it’s very important… to release stress maybe… after we talk we feel better, less stressed*” (Participant 2). The second sub-theme, ‘It helped me recognise the things that I wanted to change’ [38], was also represented with respect to giving voice to things that participants wanted to change or perceive differently in themselves and others:

“*… when I answer the question I can feel like how changing in myself… and I know about the peoples when I-I answer this question and I can like I can imagine before-before I meet the peoples how-how they group me and how I can see thems also yeah*” (Participant 3).

#### ‘Just do it’; the importance of activity

Structured activity was identified as meaningful and enjoyable, with its value closely tied to how it offers a means to support and connect with others:

“[Working in a restaurant makes me feel good about myself because] *sometime we help each other… and someone want to help and then we help yeah… the customer they after they finished eating and then we have to clean the table and then we go together and then we take the things and then we take off yeah… and it was good*” (Participant 1).

“… *sports, especially basketball because I represent my school to play other schools in high school… yeah I like to do that… with my friends… jogging… because I used to jog with my Dad*” (Participant 2).

Activities were also identified as a valuable vehicle for learning:

“*I like to go to other-other place and then I-I can see the difference between the people and them the place, how it’s look like so I can experience from them and I feel like there is, that is so good for me. I can, I can feel myself become better, and I can know about around the people*” (Participant 3).

Two participants identified their own psychological experiences as posing barriers to structured activity, for example, anxiety, anger, and low mood leading to avoidance of other people:

“*I will like, won’t bring myself there to talk to… talk to other people. I will like go to... I will go to other place to… be alone… yeah avoid*” (Participant 8);

“*I feel like, if crying, sad … can’t go out … I like to be alone, I don’t like to be sad also crying with others or be angry. Be alone is better*” (Participant 4).

One participant, however, did express the need to ‘just do it’ and continue with a feared activity in the presence of social anxiety:

“*Because when I meet the new people or the other people, sometimes so difficult for me to ask something but I always try… because when we meet the new people like we can communicate with them, we can show our confidence and we can see our-our changes in our self like*” (Participant 3).

Structural barriers were also identified, which could either prevent or complicate engagement within structured activity:

“*I wanted to be… waiter, yeah waiter… then the manager keep me to take the order and then I just take for… yeah for one time. Then after that after that he said that “I will train you to take the order again” and then… then I just wait until, until finished and then just nothing… then I was so sad*” (Participant 1).

Living in a looked after children’s home posed structural and financial barriers to activities:

“*I like go like travel... going for hike… yeah you know like cycling to the mountainside…* [but I can’t] *because I now living in the home, so we are still under care. If we finish our studies and everything and if we stepping out and then we can*” (Participant 8).

#### Motivation for change

Motivation for change – with respect to impending adulthood and a desire to engage with therapy for mental health and social problems [38] – was not reflected in the current sample. However, participants did report an enhanced sense of motivation and self-agency following completion of the social map and values assessment. Participants appeared to find the process of plotting their current social groups and valued life directions as helpful in, first, providing a starting point which could function as both an indication of what they would like to change in the future and, secondly, providing a marker against which they could subsequently compare their progress:

*“… this can help… those like me… to give motivation… to forge forward, to move forward… so thinking it is good because he need to think of what you need to do to achieve those things”* (Interpreted, Participant 1).

*“It was good because I really think like how much value, how family and other people are important to me… so it make me think of it… how important family or friends meant to you... it will affect me yes… appreciate more and spend more time with family and friends”* (Participant 2).

“*I can, I can compare like before I do somethings from beginning until I-I become like ‘I can do it’… I can know my interests like I can see like educations, and family, work. I can see three of them how they are they important in my life*” (Participant 3).

## Discussion

This pilot study assessed the feasibility and acceptability of a social recovery approach in a youth mental health setting in Malaysia. Young Malaysian participants from varied ethnic and cultural backgrounds, all of whom were vulnerable young people from low-income families, completed core social recovery assessments and discussed their experiential process reflections in a qualitative interview. Our findings suggest that, as in the UK PRODIGY trial [38, 39], the assessment of core social recovery variables appears feasible with vulnerable young people from Malay, Chinese, Indian and Indigenous populations. The time taken to administer social recovery assessments was very favourable and the mean total assessment time of just under one and a half hours is similar to what would be expected when conducted with UK participants, in English language and without interpretation. This corroborates participants’ reflections that the assessment tools were comprehensible.

The rate of completion is also favourable with a minimum completion by three quarters of the sample for the CAARMS assessment. It is notable that main reasons for non-completion related to fatigue and practical issues rather than to specific feasibility challenges presented by individual assessment tools. The present study did not allow for flexibility in dividing the assessment into multiple sessions as has been found useful in the UK context [39]. Moreover, fatigue may have been exacerbated due to the need for interpretation during assessments. In addition, current participants were not incentivised to complete assessments, i.e. there was no financial reimbursement nor potential provision of an intervention, which again may have inflated the non-completion rate.

Our findings also point towards the acceptability and the cultural validity of social recovery assessments. Participants appeared to find the assessment of time use acceptable and valuable and they engaged readily with qualitative questions around valued activities and barriers to engagement. Despite some instances of potential mild discomfort, especially relating to assessment questions about worry and anxiety, participants also valued assessments of their mental health. Participants expressed particular interest in questions about unusual experiences, such as hearing voices, with many participants suggesting a psychoeducational value to completing these assessments. Participants reported that answering assessment questions could aid in self and other reflection and help them monitor change in their emotions and experiences. Participants also expressed appreciation for the experience of reflecting on their values and social groups. For many participants, the act of completing the assessment tools appeared to give rise to an increased sense of self-agency and ability to consider and plan for a desired future. Thus, our findings suggest that the experiences of Malaysian young people echo those from our previous UK samples and perhaps underscore the intuitiveness of social recovery concepts, and the potential utility and possible universality of related clinical research tools across diverse contexts. Moreover, the essence of social recovery appeared to have some resonance for current participants insofar as they seemed to share a sense of structured activity as personally meaningful and facilitative of social connection—and reflected that engagement in such activity can be complicated by individual, psychological and systemic barriers. Our findings corroborate those of Byrne and Morrison [54], who explored participant experiences of symptom and functioning monitoring within a UK trial of early detection and prevention of psychosis, in which research engagement facilitated normalisation and ‘opening up’ around unusual psychological experiences and other difficulties. Furthermore, our findings fit with a model in which assessment itself is considered a therapeutic task rather than purely an information-gathering exercise [48].

The potential therapeutic value of being asked about unusual experiences is a particularly notable finding. Young people in Malaysia may tend to underestimate the seriousness of their own problems and set a very high threshold for help-seeking [55]. A lack of knowledge about mental health problems is considered to underpin the high level of mental health stigma in Malaysia, and education and awareness generation are therefore key activities for stigma reduction [56]. Current findings suggest that broad use of psychosis screening tools such as the PQ-16 [42] or CAARMS [57], for example in NGO or educational contexts, could in itself facilitate increased knowledge regarding unusual experiences. This could encourage engagement in mental health services at an earlier point and potentially contribute to reducing the long average DUP in Malaysia [13]. Furthermore, SRT assessment tools. Such as values-based and social group mapping exercises, could additionally provide young people with an enhanced sense of self-agency, which may also promote help-seeking and mental health service engagement.

Nevertheless, our findings also suggest that sensitivity is needed when exploring activities and engagement with family, cultural and religious groups. In asking about ‘your’ values and ‘your’ social groups there is an embedded individualism which may represent an invitation to challenge the dominant relatively collectivistic culture in Malaysia. The privileged position afforded to independence, self-enhancement, and explicit communication within Western cognitive-based therapies may also require further consideration in a Malaysian context [24–26], with reference made to locally-developed guidance around exploring spiritual or religious beliefs, resources and duties [16].

### Limitations and future research directions

Whilst efforts were made to represent young people from different ethnic and cultural communities, the qualitative methodology and small convenience sample limit the generalisability of our findings. Furthermore, no participants explicitly identified themselves as having experienced mental health problems per se. Nevertheless, current participants represented the groups that have been found to be particularly vulnerable to mental health problems in Malaysia; namely young adults from low-income families, including people from indigenous backgrounds [18]. Actual assessment scores also suggested reduced structured activity compared to the normative level in the UK [9] and revealed variance in experiences of mood, anxiety, and psychotic-like phenomena. The mean total difficulties SDQ score was in the borderline mental health problems range [58]. Moreover, the mean total PQ-16 score was just below the psychosis risk threshold [42]—with nearly half of current participants scoring in excess of this range—and one screened participant met full CAARMS criteria for at risk mental states for psychosis. Qualitative data also indicated the presence of subjective mental distress among a proportion of current participants. Additionally, for some participants, it seemed that emotional or psychological problems were preventing or reducing engagement in structured activity. Previous research has suggested that Malaysian people have limited knowledge about mental health problems, tend to underestimate their own problems and specifically do not tend to label mood and anxiety symptoms as mental health problems [55, 59]. Therefore, we cautiously suggest that our findings have relevance for young people in Malaysia experiencing mental health problems and support the acceptable use of core social recovery assessment measures within screening initiatives for early detection of young people with emerging social disability and psychological difficulties. Nevertheless, further testing in Malaysia would usefully involve young people with confirmed serious mental health problems including psychosis. Replicating the present study with a larger sample of young people would generate more robust evidence regarding the time taken to administer assessment measures. This could help to facilitate the formal translation and validation of social recovery tools in Malaysia.

A further limitation relates to the fact that the same researcher administered both assessments and interviews, with the same interpreter present, which may have impacted on responses. Identified instances of mild discomfort do nevertheless suggest that participants felt able to divulge candid reflections on the assessment process. Furthermore, whilst the present study provides preliminary evidence of feasibility and acceptability of Western assessment and therapeutic tools, their use should be further supported with an indigenisation-from-within approach. This should involve the local review of measures translated from English to consider supplementing appropriate colloquial terminology in place of explicit translations and testing the validity of these amendments. Furthermore, acceptability of measures of psychotic or psychotic-like phenomena – translated and/or locally developed – does not preclude cultural differences in the phenomenology of experiences. Measurement structures of Western constructs such as psychotic experiences may differ in a Malaysian setting [60], therefore, future research should continue to empirically explore the fit of measurement models on which Western assessments are predicated. Furthermore, explorations of intra-associations between core social recovery assessment scores, for example assessing relevant clinical time use thresholds, would also further inform a Malaysian social recovery approach. Finally, future research could evaluate practitioner perspectives on using a social recovery approach and of promoting valued structured activities with young people in Malaysia and potential for optimisation through integration with non-Western philosophies [28] – in addition to assessing potential individual and structural barriers to uptake and sustained use of screening, outcome and therapeutic formulation tools in LMIC settings.

## Conclusions

Current findings provide preliminary evidence for the ‘fit’ for the social recovery approach in a Malaysian context. In line with our work in UK settings, spending time in structured activity appeared to resonate for vulnerable young people from low-income backgrounds as personally meaningful and facilitative of social connection. These young people were able to identify individual, psychological and systemic barriers to engagement in structured activity. Furthermore, current participants appeared to value the experience of participating in social recovery assessments, including of their time use and mental health; such that the implementation of routine social recovery outcomes would appear to be of value. Furthermore, the process of delivering these assessment tools appeared feasible with respect to time taken to administer and rate of completion. Moreover, current participants seemed to find meaningful benefits in the completion of social recovery assessments; with respect to aiding in reflection on their lives and experiences and developing increased motivation and self-agency. Participants also appeared to perceive a psychoeducational benefit to being asked about their unusual psychological experiences, for example, hearing voices. The broad use of psychosis screening tools could be a valuable educational tool which could also encourage young people to seek earlier intervention.

## Data Availability

The datasets used and analysed during the current study are available from the corresponding author on reasonable request.

## Abbreviations

ARMS: At Risk Mental States
BCSS: Brief Core Schema Scales
CAARMS: Comprehensive Assessment of At Risk Mental States
DUP: Duration of Untreated Psychosis
LMI: Low and Middle-Income Country
NGO: Non-Government Organisation
PQ-16: Prodromal Questionnaire
SDQ: Strengths and Difficulties Questionnaire
SIM: Social Identity Map
SRT: Social Recovery Therapy
TUS: Time Use Survey
WHO: World Health Organisation
